# Cell-autonomous heparanase modulates self-renewal and migration in bone marrow-derived mesenchymal stem cells

**DOI:** 10.1186/1423-0127-21-21

**Published:** 2014-03-13

**Authors:** Chun-Chun Cheng, Yen-Hua Lee, Shau-Ping Lin, Wei-Chun HuangFu, I-Hsuan Liu

**Affiliations:** 1 Institute of Biotechnology, National Taiwan University, Taipei 106, Taiwan; 2 Department of Animal Science and Technology, National Taiwan University, Taipei 106, Taiwan; 3 Research Center for Developmental Biology and Regenerative Medicine, National Taiwan University, Taipei 106, Taiwan; 4 Agricultural Biotechnology Research Center, Academia Sinica, Taipei 128, Taiwan; 5 Center for Systems Biology, National Taiwan University, Taipei 106, Taiwan; 6 The Institute for Cancer Biology and Drug Discovery, Taipei Medical University, Taipei 110, Taiwan

**Keywords:** Bone marrow-derived mesenchymal stem cells, Heparan sulfate proteoglycans, Heparanase, Glycosaminoglycans

## Abstract

**Background:**

Stem cell-fate is highly regulated by stem cell niche, which is composed of a distinct microenvironment, including neighboring cells, signals and extracellular matrix. Bone marrow-derived mesenchymal stem cells (BM-MSCs) are multipotent stem cells and are potentially applicable in wide variety of pathological conditions. However, the niche microenvironment for BM-MSCs maintenance has not been clearly characterized. Accumulating evidence indicated that heparan sulfate glycosaminoglycans (HS-GAGs) modulate the self-renewal and differentiation of BM-MSCs, while overexpression of heparanase (HPSE1) resulted in the change of histological profile of bone marrow. Here, we inhibited the enzymatic activity of cell-autonomous HPSE1 in BM-MSCs to clarify the physiological role of HPSE1 in BM-MSCs.

**Results:**

Isolated mouse BM-MSCs express HPSE1 as indicated by the existence of its mRNA and protein, which includes latent form and enzymatically active HPSE1. During *in vitro* osteo-differentiations, although the expression levels of *Hpse1* fluctuated, enzymatic inhibition did not affect osteogenic differentiation, which might due to increased expression level of matrix metalloproteinase 9 (*Mmp9*). However, cell proliferation and colony formation efficiency were decreased when HPSE1 was enzymatically inhibited. HPSE1 inhibition potentiated SDF-1/CXCR4 signaling axis and in turn augmented the migratory/anchoring behavior of BM-MSCs. We further demonstrated that inhibition of HPSE1 decreased the accumulation of acetylation marks on histone H4 lysine residues suggesting that HPSE1 also modulates the chromatin remodeling.

**Conclusions:**

Our findings indicated cell-autonomous HPSE1 modulates clonogenicity, proliferative potential and migration of BM-MSCs and suggested the HS-GAGs may contribute to the niche microenvironment of BM-MSCs.

## Background

Stem cells are featured by their asymmetric behaviors of self-renewal and multipotentiality that are controlled by intrinsic genetic networks, which are modulated in response to extrinsic signals from the stem cell niches [1,2]. Stem cell niches are specialized local extracellular microenvironments that regulate stem cells to maintain tissue homeostasis and safeguards against excessive stem cell production that could lead to cancer [3]. Thus, the niche microenvironment, which may compose of various types of cells, paracrine factors, and the extracellular matrix (ECM), is one of the most important issues in stem cell biology.

In mammals, the best understood niche is hematopoietic stem cells (HSCs) in the bone marrow in which the mesenchymal stem cells (MSCs) have been suggested to contribute to the HSCs niche [4-6]. MSCs are derived from multiple developmental origins [7] and can be found all over the adult body such as bone marrow, muscle, visceral organs and adipose tissue [8-11]. Recent studies in determining the niche of bone marrow-derived MSCs (BM-MSCs) indicated that the physiological niche microenvironment of various MSCs may reside around vasculature and hence suggested that endothelial cells are part of this niche microenvironment [11,12]. The fact that transplanted bone marrow cells re-establish stem cell colony around sinusoids along with the formation of a miniature bone organ suggested that BM-MSCs share similar perivascular niche microenvironment [13]. Unfortunately, the detailed composition of this microenvironment and how the niche of mouse BM-MSCs is maintained remain elusive.

The ECM is composed of a complex mixture of fibrous proteins, polysaccharides and proteoglycans (PGs), which include a core protein and numerous covalently attached glycosaminoglycans (GAGs) [14]. Several lines of evidence indicated that sulfated GAGs in the ECM, especially heparan sulfate proteoglycans (HSPGs), modulate phenotypes of MSCs [15-18]. HSPGs, ubiquitously found in the ECM and on cell membrane of animal tissues, involve in a wide range of biological activities through their highly heterogenous HS-GAGs chains [19-21]. Accumulating evidence showed that the addition of HS-GAGs in the *in vitro* culture environment affects self-renewal and differentiation of BM-MSCs [22,23]. However, an earlier study suggested the absence of HS-GAGs in the bone marrow sinusoidal basement membrane [24]. These findings imply that the relatively low levels of HS-GAGs accumulation could be an important feature for the niche of BM-MSCs and a mechanism for the maintenance of this low HS-GAGs microenvironment must exist.

Heparanase (HPSE1) is an endo-β-glucuronidase that specifically degrades HS-GAGs and is the only known endogenous HS-GAGs degrading enzyme in vertebrates. Previous study showed that bone marrow osteoblasts express HPSE1 and ubiquitous overexpression of this gene resulted in the increase of bone mass [25,26] suggesting that osteogenesis from BM-MSCs is affected by environmental HPSE1. Furthermore, the addition of bacterial heparinase faciliated osteogenic differentiation of MSCs via BMP signaling pathway [27]. In this study, we aimed to test our hypothesis that the cell autonomous heparanase is involved in the maintenance of the niche microenvironment of BM-MSCs and exploited heparanase inhibitor, OGT2115, to study the roles of heparanase in the fate determination of mouse BM-MSCs, including differentiation, proliferation, and migration.

## Methods

### Animals

C57BL/6 mice of 6-8 weeks were purchased from the Laboratory Animal Center of Medical College in National Taiwan University (Taipei, Taiwan). Mice were kept under standard conditions, and all experimental procedures on animals were approved by the Institutional Animal Care and Use Committee (IACUC) of National Taiwan University (NTU-99-EL-87).

### Isolation of mouse BM-MSCs

Mouse BM-MSCs were harvested as previously described [28]. Briefly, bone marrow cells were cultured with four residual bone fragments together from 6- to 8-week-old C57BL/6 mice on to 60-cm^2^ tissue culture dishes (TPP, Trasadingen, Switzerland) at a density of 2 × 10^5^ cells/cm^2^ in MEM alpha (Sigma-Aldrich, St. Louis, MO, USA) supplemented with 20% fetal bovine serum (FBS; Hyclone, Logan, UT, USA), 2 mM L-glutamine (Invitrogen, Carlsbad, CA, USA), 100 U⁄mL penicillin and 100 μg/mL streptomycin (Invitrogen). The cells were incubated at 37°C in a humidified atmosphere containing 95% air and 5% CO_2_ for 72 h. The non-adherent cells were then removed by changing the medium. When cells reached 70% confluence, cells were lifted by incubation with 0.25% trypsin/0.1 mM ethylenediaminetetraacetic acid (trypsin/EDTA; Invitrogen) for 3 min at 37°C.

The BM-MSCs were enriched by negative selection. Cells were suspended in 90 μL of washing buffer per 10^7^ cells and then incubated at 4°C for 15 min on magnetic microbeads conjugated with antibodies either against CD11b or CD45 (Miltenyi Biotec, Auburn, CA, USA) according to the manufacturer’s instructions. The enriched CD11b^-^ and CD45^-^ BM-MSCs were seeded at a concentration of 5 × 10^4^ cells/cm^2^ with heparanase inhibitor OGT2115 or DMSO as vehicle control for the subsequent experiments.

### Western blotting

To evaluate the protein levels, the cells (1 × 10^6^) were washed twice with ice-cold PBS and disrupted in 200 μL of RIPA buffer (Thermo Scientific, Waltham, MA, USA). Samples were centrifuged at 14,000 *g* for 15 min, and the quantity of protein was determined by the BCA protein assay reagent (Thermo Scientific). Samples (20 μg of protein) were separated by 8% and 12% SDS-polyacrylamide gel electrophoresis (PAGE) for detecting HPSE1 and acetylated histone H3/H4, respectively and subsequently transferred onto an 0.22 μm PVDF membrane (Millipore, Billerica, MA. USA) and probed with primary antibodies which are rabbit anti-heparanase1 (Abcam, Cambridge, UK), rabbit anti-acetyl-histone H3 (Millipore) and rabbit anti-acetyl-histone H4 (Millipore). Histone H3 (rabbit anti-histone H3; Millipore) and Histone H4 (rabbit anti-histone H4; Millipore) were used as internal controls. Quantitative analysis was done by using ImageJ software (NIH) [29].

### Immunocytochemistry

After the mouse BM-MSCs were seeded onto glass coverslips for 24 hr, the cells were washed by PBS and fixed by cold methanol for 10 min at -20°C. The cells were then blocked by blocking buffer (5% BSA in PBS) and incubated with rabbit anti-heparanase 1 (Abcam) which recognizes the 65 kD precursor as well as the 50 kD and 8 kD subunits of HPSE1 at 4°C overnight. The anti-rabbit IgG conjugated Alexa-594 (Invitrogen) was used as the secondary antibody and the samples were mounted with the mounting medium containing DAPI (Abcam).

### Heparanase assay

After treated with the heparanase inhibitor (OGT2115), the extracellular composition of HS-GAGs was evaluated to test the inhibition effect of OGT2115. The proteoglycans and glycosaminoglycans from cultured cells were extracted by the extraction buffer (4 M guanidine HCl, 0.05 M Na acetate (pH = 6.0), containing 2% (w/v) Triton X-100 and protease inhibitors), and the quantities of protein were determined by the BCA protein assay reagent (Thermo Scientific). To evaluate the composition of HS-GAGs, 2 μL of sample (0.5 μg of protein) was spotted onto the 0.22 μm PVDF membrane (Millipore). After the membrane was dried, blocked by blocking buffer (5% milk and 0.1% Triton X-100 in TBS) for 1 hr, and incubated with primary antibody, mouse anti-heparan sulfate IgM (10E4, Seikagaku, Tokyo, Japan), to evaluate the complete heparan sulfate chain (10E4) content. Then chemiluminescence was performed by using goat anti-mouse IgM and IgG conjugated HRP as a secondary antibody. The signal intensity was evaluated and compared by ImageJ [29].

### Quantitative real-time reverse transcription-polymerase chain reaction (qPCR)

To evaluate the mRNA expression levels, total cellular RNA was extracted using TRIzol reagent (Invitrogen) and then treated with RNase free DNase Set (Promega, Madison, MI, USA) according to manufacturer’s instructions. Reverse transcription reactions were performed with 2 μg total RNA using the SuperScript First-Strand Synthesis System (Invitrogen), according to the manufacturer’s instructions. Real-time PCR (Bio-Rad, Hercules, CA, USA) was performed with 1 μL of the single-stranded cDNA sample with SYBR Green PCR master mix (Bio-Rad). The sequences of primers used were listed in Table 1. The qPCR program started at 95°C for 3 min followed by 40 cycles of 95°C, 10s and 60°C, 30s. Each amplification reaction was checked to confirm the absence of nonspecific PCR product by melting curve analysis. The relative gene expression level was calculated and presented with the 2^-ΔΔCt^ method. GAPDH was used as a reference gene to normalize specific gene expression in each sample.

**Table 1:** Sequences of PCR primers

	
MMP2	Forward: 5′-GGACTATGACCGGGATAAGA-3′
	Reverse: 5′-GTTGCCCAGGAAAGTGAA-3′
MMP9	Forward: 5′-CTCCAACCGCTGCATAAA-3′
	Reverse: 5′-CCCTAGGGATGCTCTCAATA-3′
MMP14	Forward: 5′-TGGCGGGTGAGGAATAA-3′
	Reverse: 5′-CTTCCTCTCGTAGGCAGTAT-3′
CXCR4	Forward: 5′-AGCTAAGGAGCATGACGGACAAGT-3′
	Reverse: 5′-AGCTAAGGAGCATGACGGACAAGT-3′
CXCR7	Forward: 5′-TTCGTGATCGGCATGATTGCCAAC-3′
	Reverse: 5′-ACTGGTTATGCTGCACGAGACTGA-3′
CXCL12	Forward: 5′-ACCCAAATGCAAAGGCTGAGTGTG-3′
	Reverse: 5′-AGCTAAGCACTGTTGCAAACCACC-3′
GAPDH	Forward: 5′-CATGGCCTTCCGTGTTCCTA-3′
	Reverse: 5′-GCGGCACGTCAGATCCA-3′
HPSE1	Forward: 5′-AAGCAGGACCGGTTGCAG-3′
	Reverse: 5′-GGTGGCCTCCTAAACTAGGG-3′

### Flow cytometric analysis

To evaluate the identity of enriched BM-MSCs, cells were immunostained with PE-conjugate monoclonal antibodies (Table 2) for 30 min at 4°C in dark according to the manufacturer’s instructions. Ten thousand cells were acquired on a Beckman Coulter FC500, and analyzed by FCS Express software (Version 4.0; Denovo software, Los Angeles, CA, USA). All experiments included negative controls that stained without antibodies and with isotype controls (eBioscience, San Diego, CA).

**Table 2:** List of antibodies used in flow cytometric analysis

**Antibody**	**Clone**	**Ref. no.**	**Conjugated**	**Isotype**	**Supplier**
CD31	390	12-0311	PE	Rat IgG2a	eBioscience
CD45	30-F11	12-0451	PE	Rat IgG2b	eBioscience
CD73	TY/11.8	12-0731	PE	Rat IgG1	eBioscience
CD105	MJ7/18	12-1051	PE	Rat IgG2a	eBioscience
Sca-1	D7	12-5981	PE	Rat IgG2a	eBioscience

### *In vitro* osteogenic differentiation

To evaluate the osteogenic differentiation potentials, BM-MSCs were cultured to near confluence and cultured in osteogenic induction medium consisting of MEM alpha (Sigma-Aldrich) supplemented with 10% FBS (Hyclone), 0.1 μM dexamethasone (Sigma-Aldrich), 10 mM β-glycerolphosphate (Sigma-Aldrich) and 50 μM ascorbic acid (Sigma-Aldrich) for 14 days [30]. The induction medium was changed every 3 days, and the bone matrix mineralization was evaluated by Alizarin red S (ARS; Sigma-Aldrich) staining. The ARS was extracted by adding 10% cetylpyridinium chloride (Sigma) in 8 mM Na_2_HPO_4_ (Merck, Darmstadt, Germany) and 1.5 mM KH_2_PO_4_ (Merck) and the absorbance was measured by SpectraMax 190 ELISA plate reader (Molecular Devices, Sunnyvale, CA, USA) at 550 nm [31].

### Cell proliferation assay

To evaluate the cell proliferation, MTT (3-(4,5-dimethyl-2-thiazolyl)-2,5-diphenyl-2H- tetrazoliumbromide) assay was performed as described previously [32]. Briefly, cells were seeded at the density of 1.5 × 10^3^ cells/well in 96 well plate and cultured without or with various concentrations (0.1, 0.4, 1 μM) of OGT2115. Cells were analyzed every two days by adding 10 μL of the MTT (5 mg/ml; Sigma-Aldrich) to each well and the cells were continued to culture for 4 hr. After the incubation, the supernatant was discarded and 100 μL of dimethyl sulfoxide (DMSO, Sigma-Aldrich) was added to each well to dissolve the formazan. The number of cells was determined according to the absorbance measured by SpectraMax 190 ELISA plate reader (Molecular Devices) at 570 nm.

### Colony formation assay

To evaluate the clonogenicity, the BM-MSCs were plated at a density of 350 cells/9.01 cm^2^ culture dish (TPP). After incubation for 9 days, the colonies formed were fixed by methanol (Sigma-Aldrich) and stained with Geimsa solution (Sigma-Aldrich) [33]. CFU numbers were enumerated by a light microscope and a cluster of at least 20 cells was defined as a CFU.

### Preparation of mouse recombinant HPSE1

To prepare the mouse recombinant HPSE1, full-length coding sequence of the gene was purchased (OriGene, Rockville, MD, USA) and subcloned into pIRES2-eGFP (Clonetech, Mountain View, CA, USA) by PCR with a FLAG-tag sequence added immediately before the stop codon to generate pHPSE1-FLAG-IRES2-eGFP. The resulted plasmid was transfected into 293T cells with TransIT-LT1 transfection reagent (Mirus Bio, Madison, WI, USA) according to the manufacturer’s instruction. The culture medium was harvested 48 to 72 hr later, reduced volume by concentrators with 10 kDa molecular weight cut-off (GE Healthcare, Pittsburgh, PA, USA) and the recombinant HPSE1 was purified with anti-FLAG M2 magnetic beads (Sigma-Aldrich) according to the manufacturer’s instruction. The buffer of the final eluent was exchanged from 0.1 M Glycine-HCl (pH 3.0) to PBS with concentrators. The resulted preparation was characterized by SDS-PAGE and western blot and the concentration was calibrated by BCA assay (Thermo Scientific).

### Transwell cell migration assay

To evaluate the role of heparanase in modulating the homing signals of BM-MSCs, 5 × 10^4^ cells were seeded on to transwells (6.5 mm, 8 μm pore; BD Biosciences, Jose, CA, USA) in MEM alpha supplemented with 1% FBS. MEM alpha with both 1% FBS and SDF-1 (200 ng/mL; R&D Systems, Minneapolis, MN, USA) was added to lower chamber. After 24 hr, non-migrating cells were wiped away slightly from the top surface of the membrane. CXCR4 inhibitor groups were pre-treated with AMD3100 (25 μg/mL; MERCK) for 1.5 hr. And the upper chamber was treated with 2 μg heparanase or 0.4 μM OGT2115. Cells migrated to the undersurface of the membrane were stained with hematoxylin (Vector Lab, Burlingame, CA, USA) and counted.

### DNA topoisomerase assay

To determine the influence of the heparanase activity on the activity of DNA topoisomerase, the nuclear protein of BM-MSCs with or without the treatment of OGT2115 was isolated and incubated with the topoisomerase I reaction buffer (500 mM Tris-Cl, 1 M KCl, 10 mM dithiothreitol, 100 mM EDTA, 50 μg/ml acetylated bovine serum albumin) and 200 ng of plasmid pUC19 at 37°C for 30 min. After incubation, the reaction contents were loaded on the 0.8% agarose gel and run for 2 to 3 hr at 5 to 10 V/cm. The sample topoisomerase activity was then relatively determined by the percentage of supercoiled plasmid.

### Statistical analysis

All experiments included at least 3 biological repeats and all values were presented as mean ± standard deviation. Statistical comparisons were analyzed with the two-tailed Student’s *t*-test or one-way ANOVA with Tukey multiple comparison. A *P*-value less than 0.05 was considered statistically significant.

## Results

### The expression of HPSE1 by mouse BM-MSCs and the enzymatic inhibition by OGT2115

**Figure 1:**
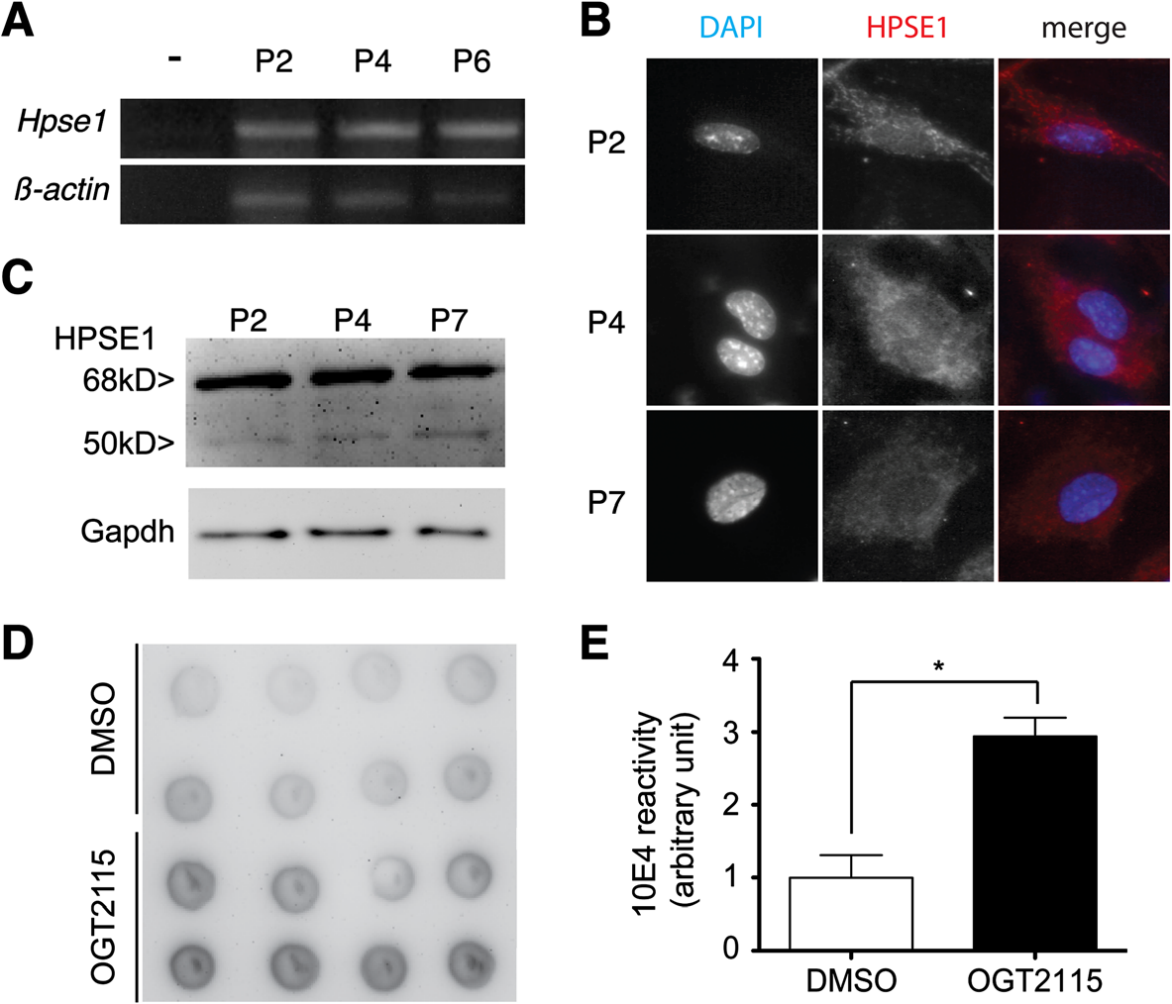
**Mouse BM-MSCs express HPSE1 and can be enzymatically inhibited by OGT2115. (A)** RT-PCR indicated the existence of mRNA of *Hpse1* in mouse BM-MSCs throughout serial passages. **(B)** Immunocytochemistry demonstrated the existence of HPSE1 protein (red) in the mouse BM-MSCs, in which the nuclei were counter-stained by DAPI (blue). **(C)** Western blot indicated that enzymatically active form (50 kDa) of HPSE1 could be detected in mouse BM-MSCs. Gapdh was used as a loading control. **(D, E)** Dot blot assay of mouse BM-MSCs demonstrated mild reactivity against 10E4 indicating a mild reservation of intact heparan sulfate glycosaminoglycans. The small molecule HPSE inhibitor, OGT2115, can repress the enzymatic activity of HPSE indicated by the statistically stronger reactivity of 10E4.

To test our hypothesis that cell autonomous heparanase participated in the maintenance of stem cell niche, we first demonstrated that mouse BM-MSCs express HPSE1. RT-PCR (Figure 1A) and immunocytochemistry (Figure 1B) showed that isolated BM-MSCs consistently express HPSE1 at mRNA and protein levels. Heparanase is translated as a pro-enzyme and requires to be processed into a smaller molecule to be enzymatically active. To clarify whether heparanase expressed by isolated BM-MSCs is enzymatically active, cell lysates were harvested for western blot. Western blot detected both intact heparanase (68 kDa) and activated heparanase (50 kDa) (Figure 1C) indicating that the BM-MSCs not only can express HPSE1 but also can activate it.

To assess the role of this enzyme in BM-MSCs, we exploited the small molecule heparanase inhibitor OGT2115 to block the enzymatic activity of heparanase. Dot-blot of cell extracts with the addition of OGT2115 showed significantly stronger reactivity against complete heparan sulfate chain (10E4) content antibody when compared to the vehicle control (Figure 1D, 1E) indicating that HPSE1 enzymatic activity in BM-MSCs was efficiently inhibited by OGT2115.

### Inhibition of HPSE did not affect molecular phenotypes and osteogenic differentiation

**Figure 2:**
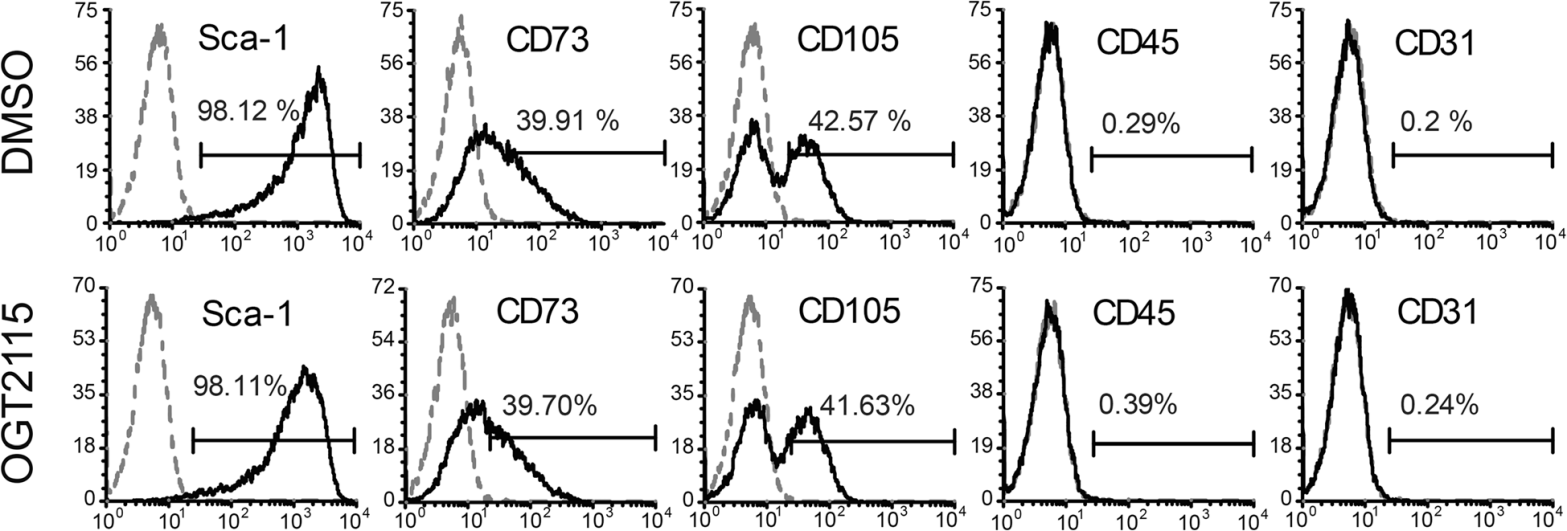
**The inhibition of cell-autonomous HPSE1 did not affect the surface marker profiles of mouse BM-MSCs.** Flow cytometric analysis of MSCs markers, Sca-1, CD73, and CD105, hematopoietic cell marker, CD45, and the endothelial cell marker, CD31 on BM-MSCs by the treatment of OGT2115 (OGT) and the DMSO control. Inhibition of HPSE did not affect these surface markers expressions on BM-MSCs.

To assess whether the inhibition of HPSE alters extrinsic signals and in turn affects stem cell property, we analyzed a panel of surface markers of mouse MSCs. Mouse BM-MSCs were positive for MSCs markers Sca-1, CD73, and CD105, while negative for the hematopoietic cell marker, CD45, and for the endothelial cell marker, CD31 (Figure 2). These expression profiles remain identical not only through serial passages at passage 2 (P2) (Figure 2), passage 4 (P4) (data not shown) and passage 7 (P7) (data not shown), but also in the presence of OGT2115 indicating that the inhibition of HPSE activity does not alter the identity of BM-MSCs.

**Figure 3:**
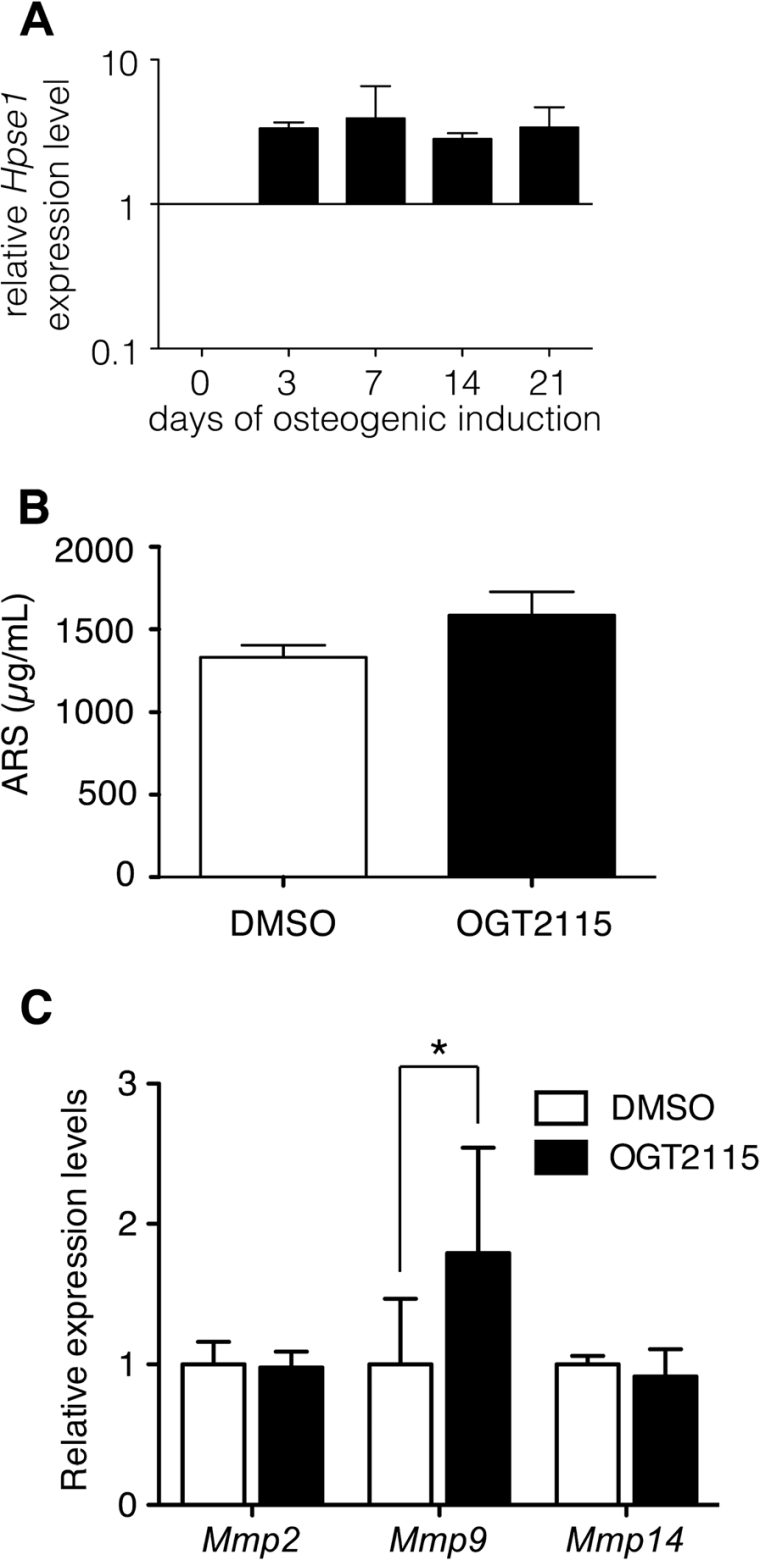
**The HPSE inhibitor OGT2115 did not affect osteogenic differentiation of mouse BM-MSCs. (A)** The expression levels of *Hpse1* increased during osteogenic differentiation. **(B)** Osteogenic potential was characterized by ARS staining after 21 days induction at the fourth passage of BM-MSCs with or without OGT2115. Quantifications of ARS staining of OGT2115 and control groups showed no difference (n = 3). **(C)** Quantitative real-time PCR analysis on the mRNA expression levels of *Mmp2*, *Mmp9* and *Mmp14* with or without the treatment of OGT2115 detected a significantly increased *Mmp9* expression when HPSE1 is enzymatically inhibited. Error bars represent standard deviation.

Previous studies indicated that environmental heparan sulfate degrading activity enhanced the osteogenic differentiation of BM-MSCs [25,27]. We therefore assessed the potential role of HPSE1 in the osteogenic differentiation of mouse BM-MSCs. In accordance with the previous results, RT-PCR showed that the *Hpse1* expression levels increased after induction for 3 days during osteogenic differentiation (Figure 3A). However, after 14 days of induction, quantitative analysis of ARS staining did not show significant difference between the control group and the OGT2115 treated group (Figure 3B) indicating that the reduction of heparanase activity does not affect the osteogenic differentiations of mouse BM-MSCs.

Previous study suggested that the increased expressions of various matrix metalloproteinases (MMPs) could compensate the loss of heparanase in genetically knockout mouse [34]. It is reasonable to speculate that at least one of the MMPs is increased in response to and compensates for the loss of the enzymatic activity of heparanase. Accordingly, we observed a significantly higher *Mmp9* expression level in HPSE-inhibited group than control group although there were no difference in *Mmp2* and *Mmp14* (Figure 3C). These data suggested that there are redundant mechanisms modulating the environmental heparan sulfate proteoglycans and the normal osteogenic differentiation of MSCs under the HPSE-inhibited condition might due to the increase of MMP9.

### Heparanase modulated cell proliferation and clonogenicity of MSCs

**Figure 4:**
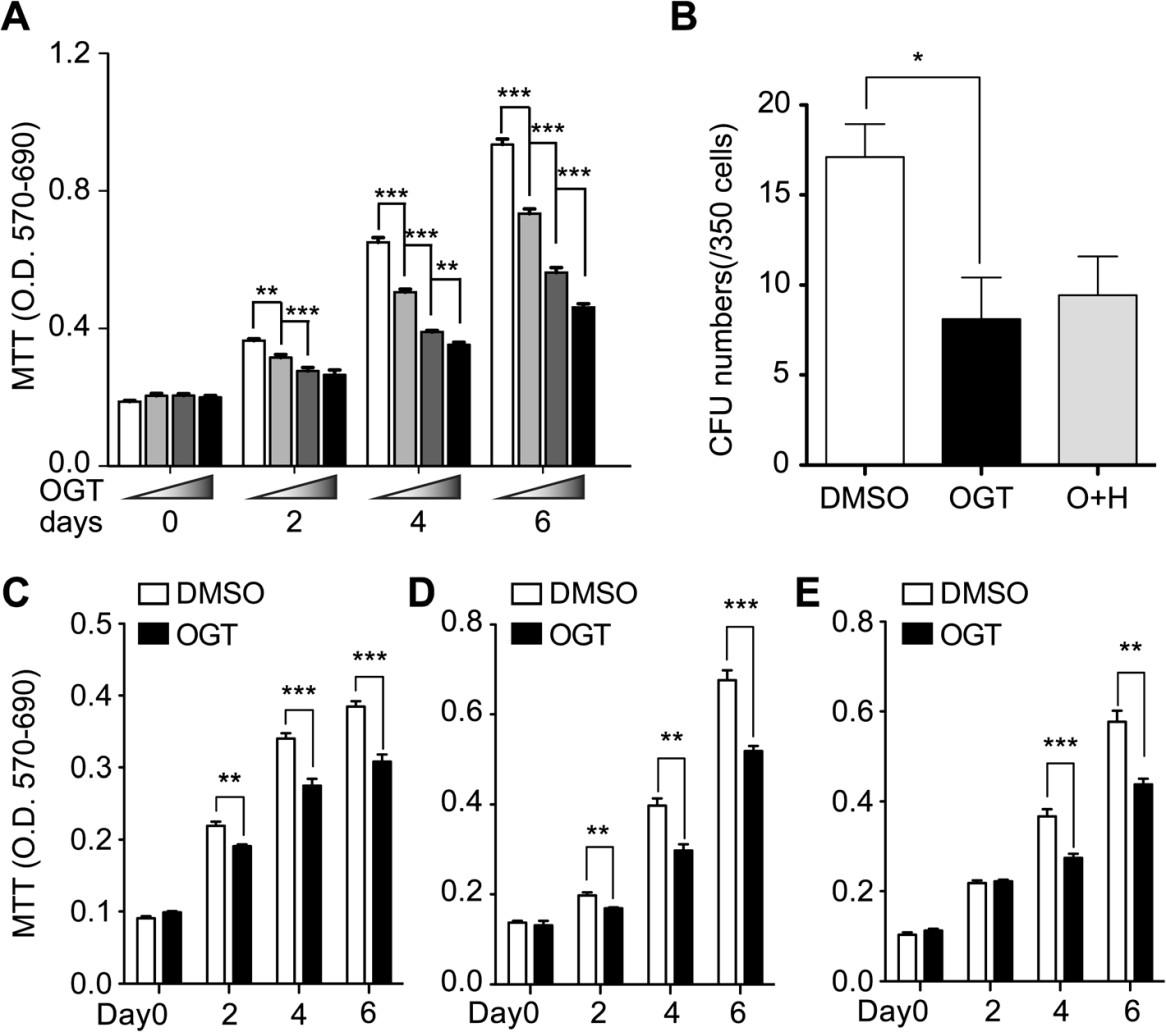
**HPSE1 promoted self-renewal of mouse BM-MSCs. (A)** Cell numbers were evaluated by MTT assay 0, 2, 4 and 6 days after the treatment of various concentrations of OGT2115. The results revealed that OGT2115 inhibited cell proliferation of BM-MSCs at fourth passage in a dose-dependent manner (n = 3). **(B)** The CFUs were significantly decreased by the treatment of OGT2115 (OGT) compared to the DMSO control, while the combined treatment of both mouse recombinant HPSE1 and OGT2115 (O + H) reversed the statistical significance (n = 3). (**C-E**) The inhibitory effect on BM-MSCs proliferation of OGT2115 was consistently reproduced at P2 **(C)**, P4 **(D)** and P6 **(E)**. *P < 0.05; **P < 0.01; ***P < 0.001.

In order to investigate whether HPSE plays a role in self-renewal and proliferation of MSCs, we first evaluated the proliferation potentials of the BM-MSCs with or without the treatment of HPSE inhibitor by MTT assay. The total cell number of HPSE-inhibited group was lower than control group in a dose-dependent fashion (Figure 4A) indicating that HPSE is important in the expansion of BM-MSCs. Since the efficiency of population expansion may correlated with the stemness of the stem cells, it is reasonable to speculate that the clonogenicity is affected by the treatment of HPSE inhibitor. We therefore assessed the population of BM-MSCs that can expand into a colony (consisted of more than 20 cells). In accordance with our speculation, HPSE-inhibited group formed significantly less CFUs than control group (Figure 4B) suggesting that HPSE is important in autonomous maintenance of cell stemness.

Since the proliferation capacity of BM-MSCs decreases along the serial passages [35,36], it is intriguing whether the effect of HPSE inhibition on the proliferation of BM-MSCs also changes. We therefore performed MTT assay on BM-MSCs 0, 2, 4 and 6 days after the treatment of HPSE inhibitor for BM-MSCs at P2 (Figure 4C), P4 (Figure 4D) and P6 (Figure 4E). The results showed that the inhibitory effect on cell proliferation could be consistently observed. Interestingly, the cell numbers began to be significantly different at day 2 in P2 and P4 BM-MSCs (Figure 4C, D), while the statistical significance were not detected until day 4 in P6 BM-MSCs (Figure 4E).

### Heparanase modulated the homing mechanism of BM-MSCs via SDF-1/CXCR4 signaling axis

**Figure 5:**
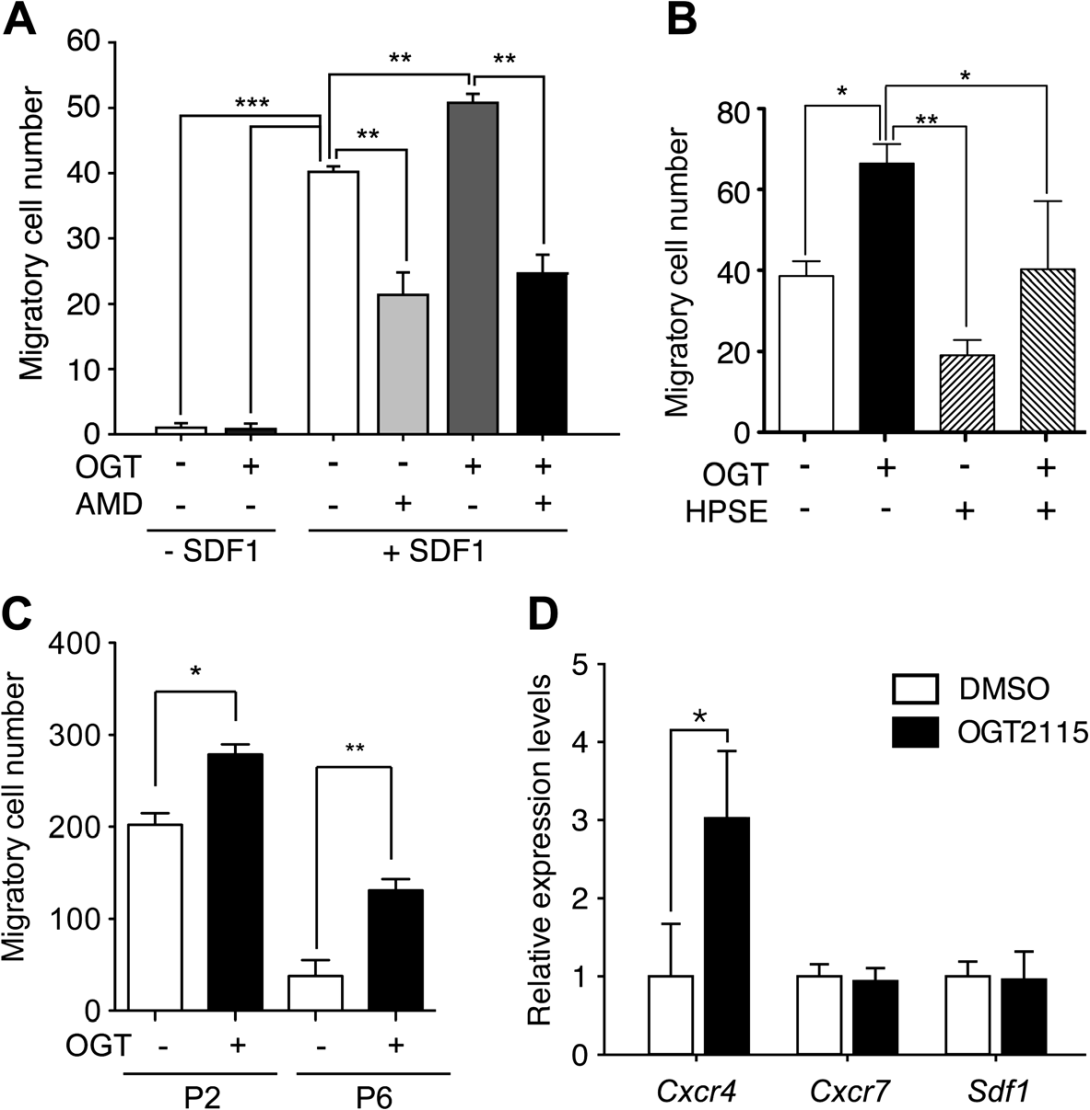
**The inhibition of HPSE activity potentiated the chemotaxis of BM-MSCs. (A)** The chemotaxis in response to SDF-1 was assayed in P4 BM-MSCs with or without the presence of OGT2115 (OGT) and AMD3100 (AMD). The BM-MSCs were strongly mobilized by SDF-1/CXCR4 signaling axis as the migratory cells were increased with the presence of SDF-1, while the presence of OGT2115 further potentiated the migration. The addition of AMD3100, the inhibitor of SDF-1/CXCR4 signaling pathway quench the migration with or without the presence of OGT2115 (n = 3). **(B)** To test the specificity of the OGT2115, the transwell migration was assayed with the presence of SDF-1. The addition of mouse HPSE1 (HPSE) demonstrated a similar trend of reduction in migratory BM-MSCs as AMD3100, while the potentiation of migration by OGT2115 can be reversed by the addition of HPSE1 (n = 3). **(C)** The potentiation of BM-MSCS migration in response to SDF-1 by the presence of OGT2115 can also be observed in P2 and P6 BM-MSCs. **(D)** qPCR analysis of migration related signals indicated that HPSE inhibitor transactivated the mRNA expression level of *Cxcr4* (n = 3). Error bars represent standard deviation. *P < 0.05; **P < 0.01; ***P < 0.001.

HSPGs modulate several signaling pathways via the binding affinity of the covalently attached HS-GAGs to a spectrum of signaling ligands and receptors [19-21,37]. It has been shown that the chemokine SDF-1/CXCR4 signaling axis plays a key role in the migration of mouse MSCs [38,39], while accumulating evidence showed that SDF-1 is modulated by HS-GAGs [18,39-42]. It is reasonable to speculate that the autonomous expression of HPSE1 also modulates the migration of mouse MSCs. We analyzed the effect of migration through inhibition of HPSE activity by using transwell assay, and the result showed that the addition of SDF-1 significantly increased the migratory cell count (Figure 5A). While OGT2115 further increased the migratory cells significantly, CXCR4 inhibitor significantly decreased the migratory cells regardless of whether HPSE was inhibited (Figure 5A). These results indicated that the inhibition of HPSE activity enhanced chemotaxis and the blocking of CXCR4 significantly decreased this chemotaxis indicating that HPSE modulates BM-MSCs homing via SDF-1/CXCR4 signaling axis.

To further demonstrate the specificity of the effect of OGT2115 on migration, the transwell migration assay with SDF-1 was repeated with or without the presence of OGT2115 and/or mouse recombinant HPSE1. In accordance with our hypothesis, the addition of mouse recombinant HPSE1 demonstrated a trend of reduced migratory BM-MSCs similarly to the CXCR4 inhibitor and significantly reversed the potentiation of migration by OGT2115 (Figure 5B) indicating that the effect of OGT2115 is specifically through the inhibition of HPSE1 and that HPSE negatively modulates the migration of BM-MSCs.

Like proliferation capacity, the migration ability of BM-MSCs also decreased along the serial passages [43]. We therefore also performed transwell migration assay with SDF-1 on P2 and P6 BM-MSCs. Consistent with the experiments done with P4 BM-MSCs (Figure 5A, B), inhibition on endogenous HPSE potentiated the cell migration at both P2 and P6 BM-MSCs (Figure 5C) indication that the effect of HPSE on modulating BM-MSCs migration persist through serial passages albeit the migration capacity decreased in later passages.

Previous studies indicated that HS-GAGs interact with SDF-1 directly and cell surface HSPGs mediate the SDF-1/CXCR4 binding and signaling [44-46]. We would like to know whether gene transactivation is also involved. To answer this question, we analyzed migration related genes including *Sdf1* (*Cxcl12*), *Cxcr7* and *Cxcr4*, and found that the expression level of *Cxcr4* increased significantly under the treatment of HPSE inhibitor (Figure 5D) suggesting that HPSE also modulates BM-MSCs via a gene transactivation mechanism.

### Heparanase participated in chromatin remodeling

**Figure 6:**
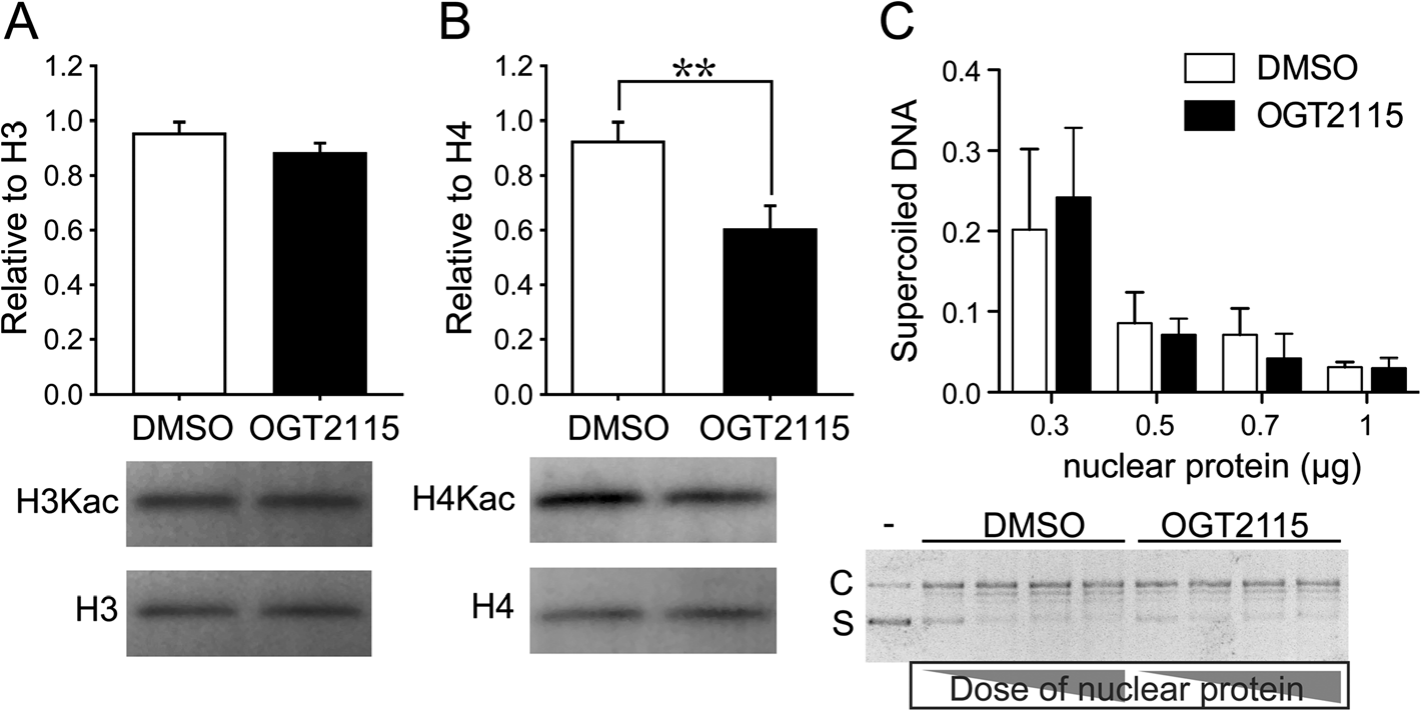
**The inhibition of HPSE decreased the acetylation level on histone H4 lysine of mouse BM-MSCs. (A)** Quantitative analysis of the acetyl-histone H3 (H3Kac) normalized by total histone H3 (H3) of BM-MSCs showed no difference in the presence or absence of OGT2115. **(B)** Quantitative analysis of the acetyl-histone H4 (H4Kac) normalized by total histone H4 (H4) of BM-MSCs. We observed significantly decreased acetylation of histone H4 with the presence of OGT2115. Error bars represent standard deviation. **(C)** Quantitative analysis of DNA topoisomerase activity with or without the treatment of OGT2115 showed similar levels of DNA topoisomerase activity. **P < 0.01 (n = 3).

Previous studies indicated that nuclear HPSE and heparan sulfate glycosaminoglycans might participate in the transcriptional regulation via the modulation of the enzymatic activities of histone acetyltrasnferases (HAT) such as p300 and DNA topoisomerase I [47-49]. Our results demonstrated the altered gene expression patterns under the inhibition of HPSE (Figures 3C and 5D). We hence hypothesized that HPSE could participate in the maintenance of self-renewal of BM-MSCs, at least partially, via this intranuclear mechanism involving in chromatin remodeling. To this end, universal histone H3- and histone H4-acetylation in BM-MSCs were quantified by western blot. Although the acetylation level of histone H3 was not altered compared to control group (Figure 6A), inhibition of HPSE activity significantly decreased the acetylation level of histone H4 (Figure 6B) suggesting cell-autonomous HPSE may participate in the biological regulation of BM-MSCs by modulating the acetylation of histone H4 and in turn the gene expression profiles. Furthermore, DNA topoisomerase assay showed similar enzymatic activities with or without the HPSE inhibition (Figure 6C) suggesting that HPSE did not affect DNA topoisomerase activity in mouse BM-MSCs.

## Discussion

In this work, the strategy of loss-of-function was undertaken to study the role of HPSE by using HPSE inhibitor, OGT2115 [50]. Previous study showed that the bone marrow stromal cells weakly express HPSE1 and this expression level is increased along with the osteogenic differentiation both *in vivo* and *in vitro*[25]. Furthermore, the observation in transgenic mouse with ubiquitous overexpression of HPSE suggested that HPSE promotes the osteogenic differentiation [25]. Similarly, we demonstrated that the isolated mouse BM-MSCs express HPSE1 throughout serial passages in the *in vitro* culture. The markedly elevated expression pattern along with the osteogenic differentiation of *Hpse1* also strongly implied that HPSE participates in the differentiation regulations of mouse BM-MSCs. Surprisingly, our results indicated that the loss of HPSE neither changed the profile of surface markers, nor affected the outcome of adipo- (data not shown) and osteo-differentiations. Interestingly, the HPSE knockout mice do not have major abnormalities probably due to the compensatory increased expression levels of matrix metalloproteinases [34]. In accordance with this finding, we also observed an increased expression level of *Mmp9* in HPSE-inhibited mouse BM-MSCs, which may provide an explanation for the lack of effect on both adipogenic and osteogenic differentiation potentials under the deficiency of HPSE activity. Since HPSE is believed to mediate many biological activities via the cleavage of the HS-GAGs attached to the core proteins of HSPGs, our finding also implies that part of the biological roles of HPSE can be achieved by the cleavage of the core proteins of HSPGs by MMPs.

Bone marrow is constituted by several types of cells including at least two populations of stem cells, HSCs and MSCs. Accumulating evidence suggested that BM-MSCs play a key role as part of the microenvironment niche for HSCs, and MSCs secreted several known HSCs regulators including SDF-1 and Wnt5a [51-53]. In contrast to what we know about the niche microenvironment of HSCs [54], little is known about how BM-MSCs maintain the self-renewal while contribute to the tissue renewal of endosteum. Due to their vicinal localization, it is reasonable to speculate the HSCs and MSCs share some regulatory mechanisms, and accordingly, both SDF-1 and Wnt5a were reported to affect both HSCs and MSCs. As a key homing regulator for HSCs [51], several transplantation studies showed that SDF-1/CXCR4 axis also play a key role in the localization of MSCs in the injured tissues [55-57]. On the other hand, although controversial cellular regulations of Wnt5a on HSCs via non-canonical pathway were reported probably due to the dose-dependent nature of Wnt ligands [52,58], it has been shown that Wnt5a promotes the osteogenic differentiation of MSCs via non-canonical pathway and antagonizes the clonogenicity supported by Wnt3a via canonical pathway [59,60]. Interestingly, HS-GAGs bind to both SDF-1 and Wnt ligands and regulate their biological activities by shaping the distribution gradients and modulating the ligand-receptor interactions [45,46,61-64]. Accordingly, a previous study suggested that the reduction in the capacity of hematopoiesis in patients received chemotherapy was due to the alteration of GAG profiles in the bone marrow, especially HS-GAGs [18]. Furthermore, ubiquitous overexpression of HPSE in transgenic mouse resulted in the increase of HSCs counts in the bone marrow [65] indicating that HS-GAGs contribute to the composition of stem cell niche microenvironment for HSCs. Although the detail mechanisms remain elusive, the HPSE secreted by marrow MSCs may modulate both MSCs and HSCs via the editing of the vicinal HS-GAGs profile.

**Figure 7:**
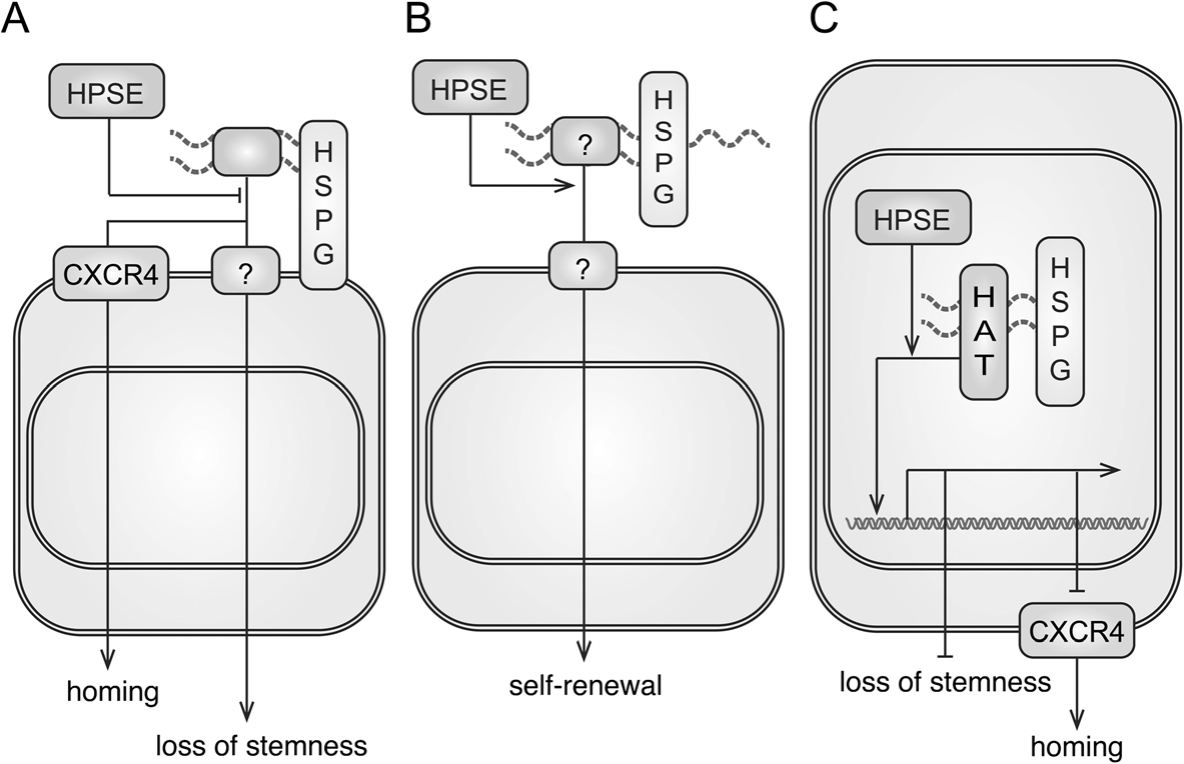
**A schematic diagram demonstrates three possible action models of HPSE in modulating BM-MSCs. (A)** HPSE could modulate putative external signals that are mediated by cell surface HSPGs and involved in MSCs self-renewal and migration such as SDF-1. **(B)** HPSE might promote the release of putative self-renewal factors that are originally trapped in the extracellular matrix HSPGs. **(C)** HPSE could be endocytosed and sorted into cell nucleus to modulate the chromatin signatures and alter the gene expression profiles such as *Cxcr4*.

Together with previous findings and our work, three models are postulated to depict the possible roles of heparan sulfate in mouse BM-MSCs (Figure 7). Firstly, HPSE might quench putative external signals that were mediated by cell surface HSPGs and could inhibit the homing, migration and self-renewal of MSCs (Figure 7A). As our data and experimental results from others, SDF-1/CXCR4 signaling axis plays a key role in MSCs homing and migration while its ligand receptor interaction is mediated by cell surface HSPGs [18,38-42]. A possible quench factor for self-renewal is FGF2 since it decreases clonogenicity of MSCs and the ligand-receptor interaction is mediated by HS-GAGs [66,67]. Secondly, heparanase might promote the release of self-renewal factors from extracellular matrix HSPGs and in turn maintain the stemness of MSCs (Figure 7B). A possible candidate is Wnt signaling since it has been reported to be involved in the stemness of MSCs [53,68] and its distribution is regulated by extracellular HS-GAGs [69]. Accordingly, β-catenin, which is the major player in the canonical pathway of Wnt signaling, was reported to transactivate the expressions of *Mmp9* and *Cxcr4*[34,70]. Thirdly, heparanase might be endocytosed and sorted into cell nucleus (Figure 7C). Numerous studies showed that the alterations on the profiles of nuclear HS-GAGs modulate chromatin-remodeling factors such as histone acetyltransferases (HATs) [47,71,72]. In accordance with these results, we observed the alteration in the acetylation levels of histone H4 under the treatment of heparanase inhibitor. Therefore, this is also a potential hypothesis that HPSE1 secured the HATs activities and in turn a set of self-renewal promoting genes were transactivated to maintain the stemness of MSCs.

## Conclusion

In this study, we demonstrated that mouse BM-MSCs autonomously express HPSE1. Loss of HPSE activity did not result in the alteration of phenotypes of BM-MSCs as well as the osteogenic differentiation. It is possible that the increased expression of *Mmp9* compensates for the loss of HPSE activity. We found that loss of HPSE activity decreased self-renewal and proliferation of BM-MSCs. Moreover, HPSE regulated the migration of BM-MSCs by modulating SDF-1/CXCR4 signaling axis. Furthermore, HPSE participated in the modification of histone H4 acetylation in the nucleus of BM-MSCs. Together, these findings suggest that cell-autonomous HPSE1 modulates vicinal and nuclear HS-GAGs profiles of MSCs and in turn participates the regulation of MSCs biology.

## Abbreviations

BM-MSCs: Bone marrow-derived mesenchymal stem cells; HS-GAGs: Heparan sulfate glycosaminoglycans; HPSE: Heparanase; MMP: Matrix metalloproteinase; ECM: Extracellular matrix; HSCs: Hematopoietic stem cells; PGs: Proteoglycans; GAGs: Glycosaminoglycans; HSPGs: Heparan sulfate proteoglycans; HATs: Histone acetyltransferases.

## Competing interests

The authors declare that they have no competing interests.

## Authors’ contributions

CCC and YHL performed all of the experiments in this study and contributed to the data analysis and interpretation. SPL and WCH contributed to the experimental design of this study. IHL participated in the conception, experimental design, and data interpretation of this study. All authors participated in the drafting of this manuscript. All authors read and approved the final manuscript.
